# Exploring the impact of Chinese medicine on CD4 cell counts in people living with HIV by mixed-effects model: a retrospective cohort study based on propensity score matching

**DOI:** 10.3389/fimmu.2026.1863625

**Published:** 2026-09-24

**Authors:** Runyu Zhu, Yantao Jin, Qiujia Kang, Yanmin Ma, Huangchao Jia, Jun Yuan, Xiuxia Ma, Lihua Chen, Ruohan Sun, Zichen He, Siyu Wang, Juan Wang, Liran Xu, Huijun Guo, Qianlei Xu

**Affiliations:** 1 Department of Acquired Immune Deficiency Syndrome Treatment and Research Center, First Affiliated Hospital of Henan University of Chinese Medicine, Zhengzhou, China; 2 The First Clinical Medical School, Henan University of Chinese Medicine, Zhengzhou, China; 3 Henan Key Laboratory of Viral Diseases Prevention and Treatment of Chinese Medicine, Henan University of Chinese Medicine, Zhengzhou, China; 4 Center for AIDS/STD Control and Prevention, Center for Disease Control and Prevention of Henan Province, Zhengzhou, China

**Keywords:** CD4 cell counts, Chinese medicine, HIV, mixed effect model, real world study

## Abstract

**Background:**

CD4 cells are the targets of human immunodeficiency virus (HIV). If left untreated, HIV enters CD4 cells and replicates, ultimately leading to the death of the infected cells. The loss of these cells impairs immune function. As Chinese medicine (CM) can be used to treat people living with HIV (PLHIV), it is important to explore the long-term effects of CM on the CD4 cell count of PLHIV.

**Methods:**

An open-label study was conducted using data from conventional medical records. PLHIV who met the criteria and were treated with CM (CM group) and not treated with CM (non-CM group) were propensity-score matched in a 1:1 ratio and followed for 10 years; their CD4 cell counts were collected at baseline and every year thereafter. Linear mixed-effects models (LMEMs) were used to assess the effects of CM on the CD4 cell count, and subgroup and sensitivity analyses were performed to provide further insights.

**Results:**

A total of 1,908 PLHIV were enrolled in this study. No differences in baseline characteristics were observed between the CM and non-CM groups. Univariate LMEM analysis showed that CD4 cell count increased significantly more in the CM group than in the non-CM group (estimate and 95% CI: 1.33; 0.84–1.81; *P* < 0.05). Multivariate LMEM analysis also demonstrated a similar trend (estimate and 95% CI: 2.97; 1.91–4.02; *P* < 0.05). Subgroup analysis indicated that CM supported higher CD4 cell counts across almost all subgroups and particularly among women and those with lower baseline CD4 cell counts. Subsequent sensitivity analyses further confirmed the positive effects of CM on CD4 cell count among PLHIV.

**Conclusions:**

CM might be associated with maintaining higher CD4 counts on top of existing ART regimens, especially in those with a lower baseline CD4 cell count.

## Introduction

Acquired immunodeficiency syndrome (AIDS) induced by human immunodeficiency virus (HIV) continues to pose a global public health threat. According to the report of UNAIDS, there were approximately 40.8 million people living with HIV (PLHIV) worldwide by the end of December 2024 (1). CD4 cells are the primary target cells for HIV. If left untreated, HIV enters these cells, replicates within them, and ultimately leads to their demise. The resultant CD4 cell depletion compromises immune function, which is associated with various adverse clinical manifestations such as opportunistic infections (2), tuberculosis (3), and malignant tumors (4). CD4 cell count serves as a crucial indicator of disease progression and plays a pivotal role in guiding medical therapy (5), particularly in resource-limited settings where viral load monitoring may be restricted (6).

The introduction of combination antiretroviral therapy (ART) has brought great hope for PLHIV, as it inhibits HIV replication and increases CD4 cell counts, thereby preserving or restoring immune function. While ART provides substantial benefits for PLHIV, some individuals experience limited improvement in their CD4 cell count (7). Chinese medicine (CM) can also play a crucial role in AIDS treatment, particularly in China, by prolonging survival, improving quality of life, and increasing CD4 cell count among PLHIV (8–11). In recent years, with the development of AIDS management models, in addition to traditional complementary therapies such as CM, the optimization of ART treatment has continuously improved the simplicity of the treatment plan to reduce the burden on patients and has increasingly focused on the treatment of patients with mental health disorders. Simultaneously, patient medication compliance and standardized drug management have increasingly become important parts of AIDS management (12–14). Previous studies on the effect of CM on CD4 cell count were primarily cross-sectional or short-term cohort studies with small sample sizes (15–17). To validate their findings, we previously designed a 14-year cohort study in which we qualitatively observed that CM can enhance CD4 counts among PLHIV with baseline CD4 counts below 350 cells/μL (9). To provide further evidence supporting these results, we designed this study to quantitatively explore the long-term impact of CM on CD4 cell counts. We used the mature extension package “nlme” in R (18) to construct the LMEMs and employed the maximum likelihood (ML) method to estimate model parameters to compare the fixed effects of CM (19) and to quantitatively explore the long-term impact of CM on CD4 cell counts.

## Methods

### Study setting and design

Since 2004, the National Administration of Traditional Chinese Medicine has sponsored a national CM–AIDS Treatment Trial Program (NCMAIDSTP) for PLHIV. All individuals voluntarily chose to participate in the NCMAIDSTP and provided signed informed consent. In the NCMAIDSTP of Henan Province, participants received the Yiaikang capsule, a Chinese medicine. Each capsule contains 0.5 g. The approval number is Yu Yao Zhi Zi Z20150001 (Zheng). The ingredients include *Dioscoreae Rhizoma*, *Jujubae Fructus*, *Ginseng Radix et Rhizoma*, *Astragali Radix*, *Coicis Semen*, *Atractylodis Macrocephalae Rhizoma*, *Poria*, *Zingiberis Rhizoma*, *Angelicae Sinensis Radix*, *Chuanxiong Rhizoma*, *Paeoniae Radix Alba*, *Rehmanniae Radix*, *Ophiopogonis Radix*, *Asini Corii Colla*, *Bupleuri Radix*, *Cinnamomi Ramulus*, *Platycodonis Radix*, *Saposhnikoviae Radix*, *Scutellariae Radix*, *Glycyrrhizae Radix et Rhizoma*, *Sojae Semen Germinatum*, and *Ampelopsis Radix* (20). Five capsules were administered orally to participants three times each day. Participants were required to have their medical information recorded on a monthly basis. The NCMAIDSTP of Henan Province was reported elsewhere (21). Owing to historical reasons, there was a large concentration of PLHIV residing in rural areas of Henan Province (22), resulting in a relatively short NCMAIDSTP enrollment period in affected areas. In 2004, 2006, 2009, and 2012, groups of PLHIV were enrolled in the NCMAIDSTP of Henan Province. Based on that work, we conducted an open-label, 10-year retrospective cohort study, with the NCMAIDSTP initiation time serving as the starting point of the cohort and the PLHIV who participated in the NCMAIDSTP deemed the CM group. PLHIV in the same region during the same period who did not participate in the NCMAIDSTP formed the control group, referred to as the non-CM group.

### Study population

The study population comprised PLHIV who were residing in the areas of Henan Province where the NCMAIDSTP was conducted and who met the following criteria. Participants comprised individuals with HIV infection confirmed by Western blotting between October 2000 and the beginning of the cohort study, aged 18–60 years, and with a CD4 cell count less than 350 cells/μL at baseline. Individuals with incomplete baseline data or a CD4 cell count recorded less than twice over the 10-year follow-up period were excluded from the study.

PLHIV meeting the criteria were first identified and then individuals in the CM and non-CM groups were matched by propensity score (PS) at a 1:1 ratio. The non-CM population could have overlapped with different cohort start times, so it was matched in chronological order based on start year (2004, 2006, 2009, or 2012), and each study participant was included only once.

### Data collection and variables

Individual data—which included age, sex, marital status, ethnicity, education, occupation, route of infection, date of confirmed HIV infection, date of ART commencement, date of death, and date of censoring—were acquired from the conventional medical records of the National Free Antiretroviral Treatment Program (NFATP), which was managed by the Chinese Center for Disease Control and Prevention and did not interfere with NCMAIDSTP. The CD4 cell counts and dates were obtained from both registries. Data on CM administration were obtained from the conventional medical records associated with the NCMAIDSTP. The CD4 cell counts analyzed in this study were those recorded closest (within 6 months) to the annual anniversary date of the cohort. If no record was entered during this interval, the variable was defined as missing.

The effect of CM was evaluated on top of the PLHIV’s ongoing ART. In the free ART program, the treatment plan is determined based on the individual condition of the PLHIV and the available ART drugs in China at present. All first-line therapy includes two nucleoside reverse transcriptase inhibitors (NRTIs) and one non-nucleoside reverse transcriptase inhibitor (NNRTI); the second-line therapy consists of one protease inhibitor (PI)/integrase inhibitor (INSTI) and two NRTIs (23). With the update of the National Free AIDS Antiviral Drug Treatment Manual in China, the specific usage and dosage are not fixed and will change accordingly.

### Data analysis

The PS was calculated using a multivariable logistic regression model with the group as the dependent variable and baseline variables such as age, sex, marital status, education, route of infection, duration of HIV diagnosis, ART duration, and baseline CD4 cell count as independent variables. Matching of the CM and non-CM groups at a 1:1 ratio was performed using the nearest-neighbor method with a fixed caliper width of 0.01.

Demographic and clinical data were compared after matching between PLHIV in the CM and non-CM groups. Continuous variables are presented as the mean with interquartile range (IQR) and compared using the paired t-test. Categorical variables are expressed as numbers with percentages and compared using Fisher’s exact or the chi-squared test. LMEMs were used to assess the effect of CM on the CD4 cell count by controlling for other variables. Normality tests were conducted to assess the assumptions of the LMEMs. Time, group, sex, age (<40 years, 40–50 years, or >50 years), marital status, education, duration of HIV diagnosis (<1 year, 1–3 years, 3–5 years, or >5 years), ART status, and baseline CD4 cell count (cells/μL) were independent variables. The IDs of PLHIV were taken as a random intercept and time as random slope. Estimates with 95% confidence intervals (CIs) generated by the LMEMs were used to determine the factors associated with CD4 cell count. We compared the merits of the models using the Akaike information criterion (AIC), Bayesian information criterion (BIC), and log-likelihood (logLik), and we conducted likelihood ratio tests on the models to determine the optimal model. The diagnosis of the goodness of fit of the model was based on four aspects: residual random diagnosis, residual normality diagnosis, random-effect normality diagnosis, and collinearity diagnosis. Multiple imputation (Classification and Regression Trees, CART) and random forest imputation were used to fill in the missing on CD4 cell count during the follow-up period, which were then analyzed by the optimal multivariate LMEM. Among them, multiple imputation was used to generate 50 imputed datasets, which were fitted by the optimal multivariate LMEM separately, and the results were aggregated using Rubin’s rules. All of the data were organized and analyzed using R version 4.5.0. The R packages used include “tidyverse,” “ggplot2,” “patchwork,” “nlme,” “emmeans,” “sjPlot,” “performance,” etc. A difference is considered statistically significant if *P <*0.05.

## Results

### Participant characteristics

A total of 10,049 PLHIV who met the inclusion criteria were included in this study. After 1:1 PS matching, 2,112 individuals were selected. Of these, 204 individuals with fewer than two CD4 cell count measurements during the 10-year follow-up were excluded, resulting in a final sample of 1,908 individuals (**Figure 1**). The average follow-up time for the CM group was 8.05 years (SD, 3.01), which was longer than that of the non-CM group (8.01 years; SD, 2.93) (*P <*0.05). There were 248 deaths in the CM group, with a 10-year mortality rate of 25%, which was lower than that of the non-CM group (243 deaths, 10-year mortality rate of 26.5%) (*P <*0.05). No significant differences were observed between the baseline characteristics of the CM and non-CM groups (*P >*0.05). The baseline characteristics are summarized in **Table 1**.

**Figure 1:**
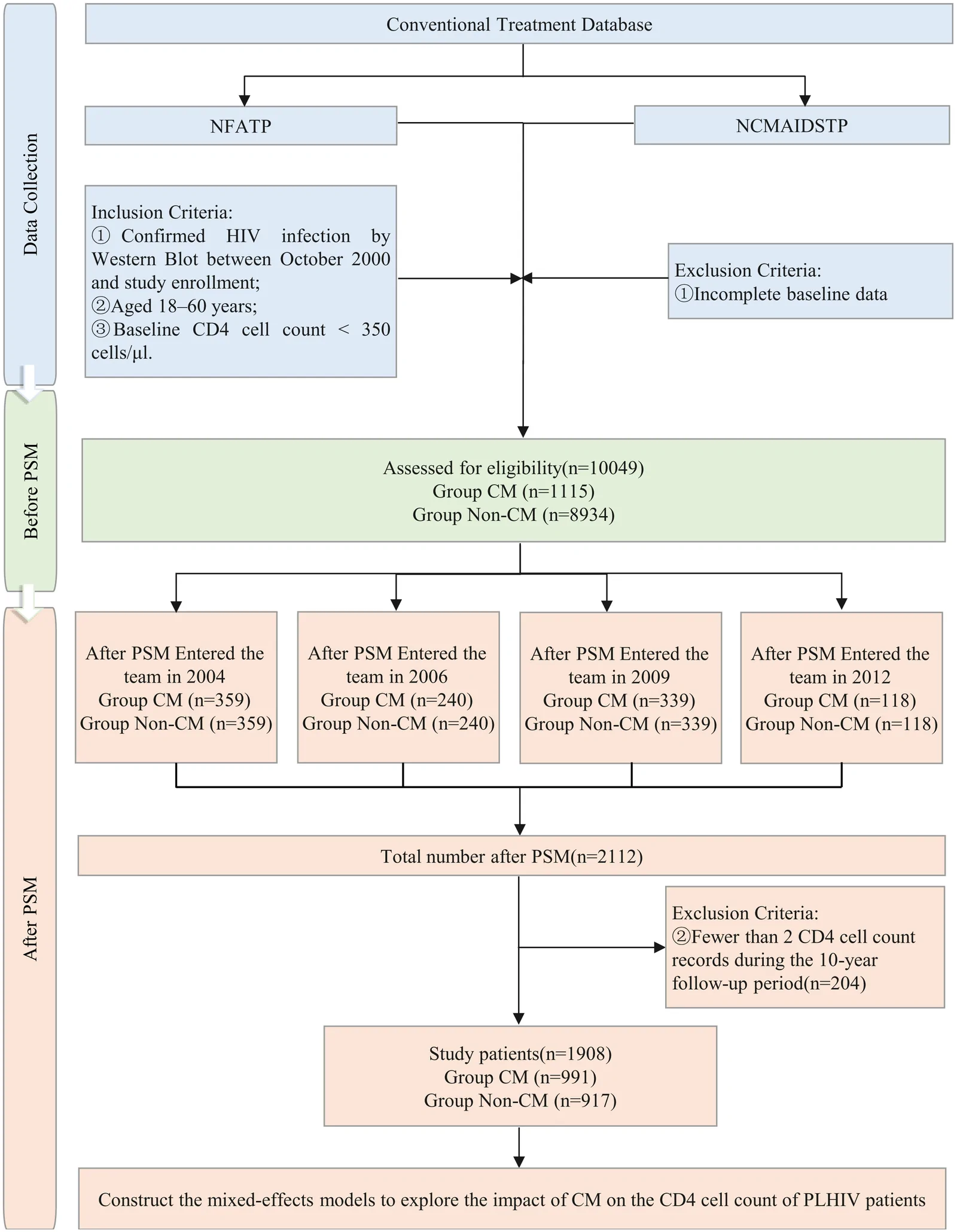
Study population screening flowchart. CM, Chinese medicine; NFATP, National Free Antiretroviral Treatment Program; NCMAIDSTP, national CM–AIDS Treatment Trial Program; non-CM, non-Chinese medicine; PSM, propensity-score matching.

**Table 1:** Comparison of the baseline characteristics of PLHIV in the non-CM and CM groups after PSM.

Variables	Total (n = 1,908)	Non-CM (n = 917)	CM (n = 991)	*P*-value
Sex				0.562
Female, n (%)	963 (50.5%)	456 (49.7%)	507 (51.2%)	
Male, n (%)	945 (49.5%)	461 (50.3%)	484 (48.8%)	
Age (years), Mean (Q1, Q3)	44.3 (39, 50)	44.4 (39, 50)	44.2 (39, 50)	0.612
Marital status				0.632
Married, n (%)	1421 (74.5%)	688 (75.0%)	733 (74.0%)	
Sig/window, n (%)	487 (25.5%)	229 (25.0%)	258 (26.0%)	
Ethnicity				0.346
Han, n (%)	1,898 (99.5%)	914 (99.7%)	984 (99.3%)	
Others, n (%)	10 (0.52%)	3 (0.33%)	7(0.71%)	
Occupation				0.486
Farmer, n (%)	1,867 (97.9%)	900 (98.1%)	967 (97.6%)	
Others, n (%)	41 (2.15%)	17 (1.85%)	24 (2.42%)	
Educational status				0.944
≤6 years, n (%)	1,173 (61.5%)	565 (61.6%)	608 (61.4%)	
>6 years, n (%)	735 (38.5%)	352 (38.4%)	383 (38.6%)	
Route of infection				1.000
Plasma, n (%)	1,709 (89.6%)	821 (89.5%)	888 (89.6%)	
others, n (%)	199 (10.4%)	96 (10.5%)	103 (10.4%)	
Duration of HIV diagnosis (years), Mean (Q1, Q3)	2.86 (0, 5)	2.90 (0, 5)	2.83 (0, 5)	0.570
ART duration (year), Mean (Q1, Q3)	2.20 (1, 4)	2.22 (1, 4)	2.19 (1, 4)	0.704
baseline CD4 cell count (cells/μL), Mean (Q1, Q3)	211 (146, 286)	212 (150, 283)	209 (142.5, 288)	0.473

ART, active antiretroviral therapy; CM, Chinese medicine; HIV, human immunodeficiency virus; PLHIV, people living with HIV; PS, propensity score.

### Annual CD4 cell count on anniversary date

During the 10 years of follow-up, the CD4 cell counts of the PLHIV in both groups increased yearly. The CD4 cell counts of the CM group increased more than those of the non-CM group in the first 5 years, and the differences were significant (*P* < 0.05). The detailed changes in the annual CD4 cell counts and the statistical comparisons between the two groups are shown in **Figure 2**.

**Figure 2:**
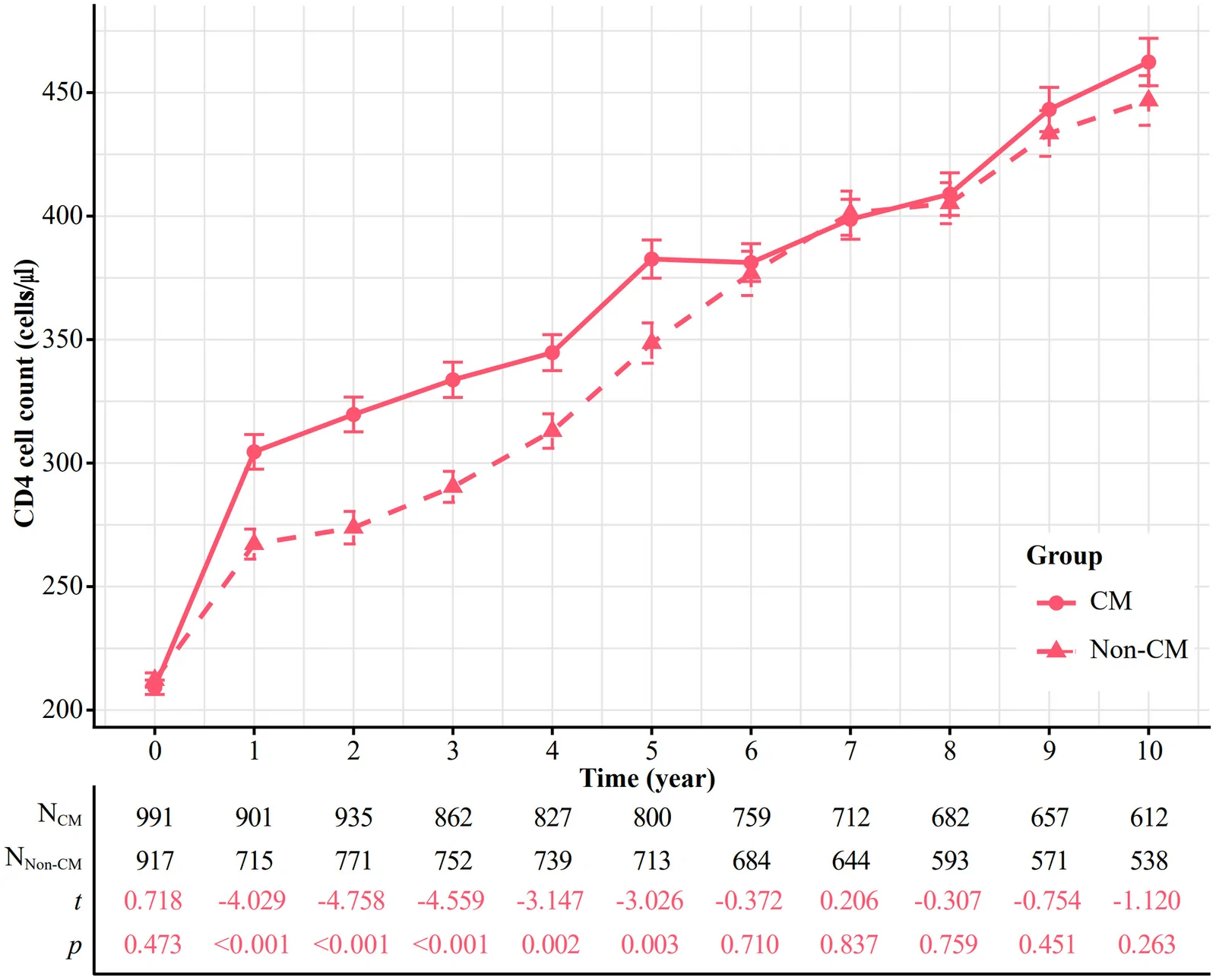
Annual CD4 cell counts in the CM and non-CM groups.

### Data normality test

In accordance with the assumptions of the LMEM, normality tests were conducted on the repeated CD4 cell count measurements. The original data violated the normality assumption (**Supplementary Figures S1A, C**), whereas square root transformation yielded a distribution that more closely approximated normality (**Supplementary Figures S1B, D**). Consequently, the square root of the CD4 (Sqrt of CD4 cell count) was used as the dependent variable in the LMEM.

### Construction and screening of the univariate LMEMs

Taking the Sqrt of CD4 cell count as the dependent variable, individual ID as the random intercept, and group and time as fixed effects, we constructed the model *RIAS* with interaction terms and random slopes. We also constructed model *AR1* with increased autocorrelation structure, model *Var* with increased heteroscedasticity structure, model *ns* with numerical non-linear structure, and model *all*, which includes the *AR1*, *Var*, and *ns* structures (**Table 2**). Likelihood ratio tests and comparisons of the AIC, BIC, and logLik values all indicated that the model *all* had the best fit (**Table 3**).

**Table 2:** The composition and interpretation of the univariate linear mixed effects models.

Name	Model	Explanation
*RIAS*	lme(Sqrt(cd4)∼group*time,random = ∼time|ID, method = “ML”, data = data)	Interaction term + random intercept + random slope
*AR1*	lme(Sqrt(cd4)∼group*time,random = ∼time|ID, method = “ML”, correlation = corAR1(form = ~ time|ID),data = data)	Add autocorrelation structure
*Var*	lme(Sqrt(cd4)∼group*time,random = ∼time|ID, weights = varIdent(form = ∼ 1 | group),method = “ML”, data = data)	Add heteroscedastic structure
*ns*	lme(Sqrt(cd4)∼factor(time) * group, data = dat1,random = ∼1 + ns(as.numeric(time), df = 2) | ID,method = “ML”, data = data)	Adding non-linear structure
*all*	lme(Sqrt(cd4)∼factor(time) * group, data = dat1,random = ∼1 + ns(as.numeric(time), df = 2) | ID,correlation = corAR1(form = ∼time|ID),weights = varIdent(form =∼1 | group),method = “ML”, data = data)	Add autocorrelation structure + heteroscedastic structure + non-linear structure

**Table 3:** Comparison of the univariate linear mixed effects models.

Model	AIC	BIC	logLik	L.Ratio	*P*-value
*RIAS*	85,950.9	86,011.5	−42,967.4	–	–
*AR1*	85,686.5	85,754.7	−42,834.2	266.4	<0.001
*Var*	85,854.5	85,922.7	−42,918.3	98.4	<0.001
*ns*	85,723.4	85,928.0	−42,834.7	265.5	<0.001
*all*	85,500.3	85,598.8	−42,737.1	460.6	<0.001

L.Ratio and *P*-value are for comparisons with the model *RIAS*.

### Diagnostic and result analysis of optimal univariate model

The diagnostic plots demonstrated that the model *all* has a good fit (**Figures 3A–C**). The coefficient CI graph indicates a significant difference between the groups (**Figure 3D**). By comparing the predicted values of the univariate optimal model with the annual trends of the original data, we observed that the Sqrt of CD4 cell counts in both the CM and non-CM groups showed a gradually increasing trend over the years. The CM group trend curve was consistently higher than that of the non-CM group, suggesting better overall immune recovery in the CM group (**Figure 3E**). Analysis of the univariate optimal model results also showed that CD4 cell counts increased more in the CM group than in the non-CM group (estimate and 95% CI: 1.33, 0.84–1.81; *P* < 0.05). Sex, age, marital status, education, duration of HIV diagnosis, ART status, and CD4 cell count at baseline were used separately to replace the grouping in the optimal univariate model to account for other factors that would affect the Sqrt of CD4 cell count. CD4 cell counts increased over time, with greater increases observed in PLHIV with longer HIV infection duration, those who were female, those who received ART, and those with higher baseline CD4 cell counts (*P* < 0.05) (**Figure 3F**).

**Figure 3:**
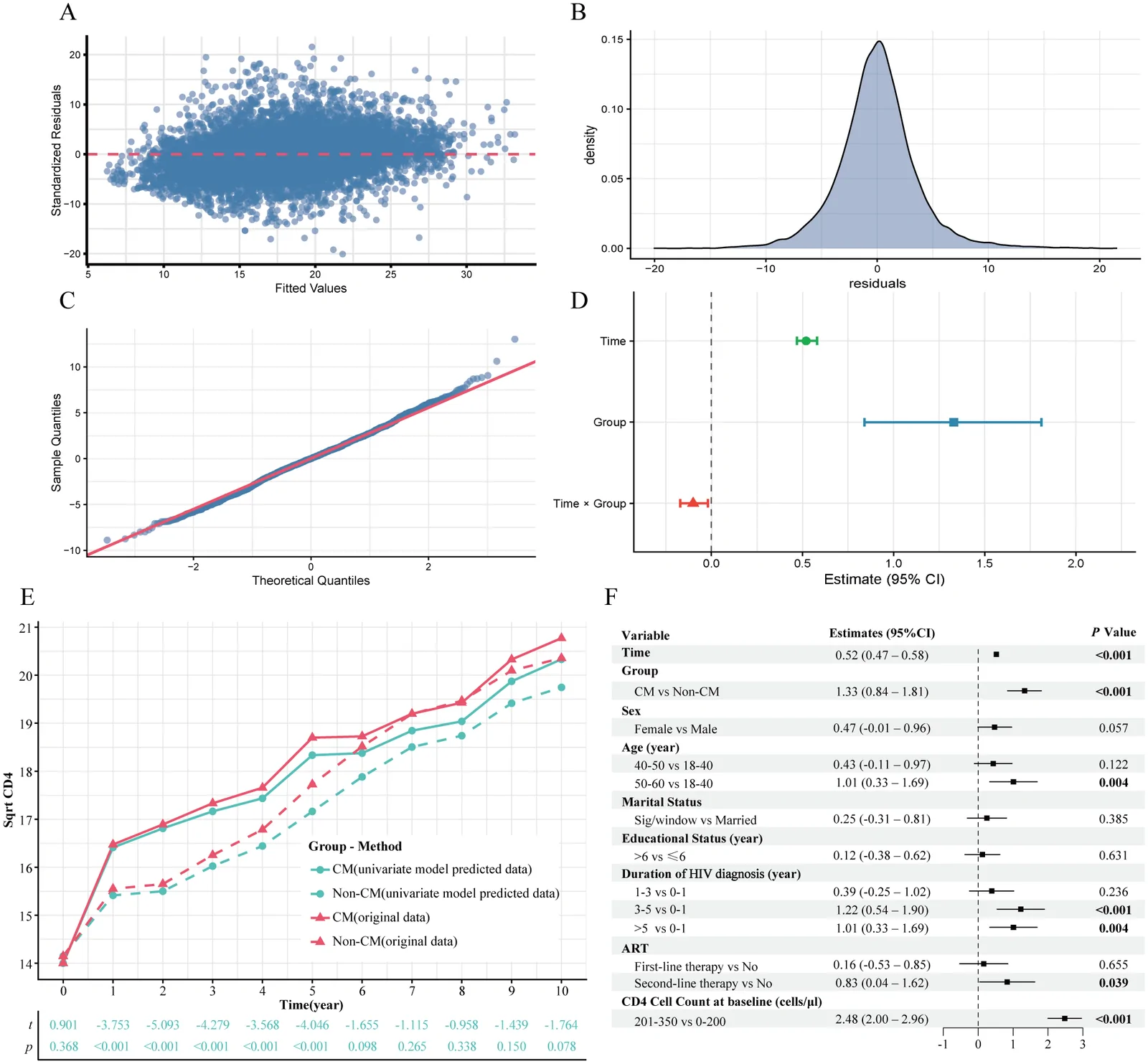
Diagnostic analysis and results of the univariate optimal mixed model. **(A)** the linear relationship diagnostic plot, **(B)** the normality plot for random effects, **(C)** the normality diagnosis of residuals and the normality quantile–quantile plot, **(D)** the optimal model effect plot, **(E)** the Sqrt of CD4 cell count annual trend plot, and **(F)** the results of the optimal univariate model.

### Construction and screening of the multivariate LMEMs

Based on the screening results of the univariate model, variables with statistically significant effects (*P <*0.05) were included in the multivariate LMEM. Additionally, considering potential dynamic changes in these variables over time or between groups, interaction terms between each variable and time, as well as between each variable and group, were incorporated into the analysis. We constructed the model *RIAS_full* by taking the Sqrt of CD4 cell count as the dependent variable; time, group, sex, ART status, age, CD4 cell count at baseline, and their interactions with time and group as fixed effects; individual ID as the random intercept; and time as the random slope. Three models were also constructed: model *AR1_full* with an increased autoregressive structure, model *Var_full* with an increased heteroscedastic structure, and model *ns_full* with an increased numerical non-linear structure; we further constructed model *all_full*, which included the three increased structures (**Table 4**). Through likelihood ratio tests and comparisons of the model AIC, BIC, and logLik values, the model *all_full* was determined to be the optimal model (**Table 5**).

**Table 4:** The composition and interpretation of the multivariate linear mixed effects models.

Name	Model	Explanation
*RIAS* *_full*	lme(cd4a~factor(time)*group+sex+age+age:time+poib+ART+cd4b+cd4b:time,random = ~time|ID, method = “ML”, data = data)	Interaction term + random intercept + random slope
*AR1_full*	lme(cd4a~factor(time)*group+sex+age+age:time+poib+ART+cd4bcat+cd4b:time, random = ~time|ID, correlation = corAR1(form = ~ obstime|ID), method = “ML”, data = data)	Add autocorrelation structure
*Var_full*	lme(cd4a~factor(time)*group+sex+age+age:time+poib+ART+cd4b+cd4b:time, random =~time|ID, weights = varIdent(form = ~ 1 | group), method = “ML”, data = data)	Add heteroscedastic structure
*ns_full*	lme(cd4a~factor(time)*group+sex+age+age:time+poib+ART+cd4b+cd4b:time,method = “ML”, random = ~ 1 + ns(as.numeric(time), df = 2) | ID, data = data)	Adding non-linear structure
*all_full*	lme(cd4a~factor(time)*group+sex+age+age:time+poib+ART+cd4b+cd4b:time+sex:group+cd4b:group+ART:group,random = ~ 1 + ns(as.numeric(time), df = 2) | ID,correlation = corAR1(form = ~ time|ID),weights = varIdent(form = ~ 1 | group), method = “ML”, control = ctrl, data = data)	Add autocorrelation structure + heteroscedastic structure + non-linear structure

**Table 5:** Comparison of the multivariate linear mixed effects models.

Model	AIC	BIC	logLik	L.Ratio	*P*-value
*RIAS_full*	85,750.7	85,902.3	−42,620.6	–	–
*AR1_full*	85,500.5	85,659.7	−42,729.2	252.2	<0.001
*Var_full*	85,648.1	85,807.2	−42,803.0	104.6	<0.001
*ns_full*	85,524.6	86,002.1	−42,699.3	312.1	<0.001
*all_full*	85,268.8	85,488.6	−42,605.4	499.9	<0.001

L.Ratio and *P*-value are for comparisons with model *RIAS_full*.

### Diagnostic analysis and results of the optimal multivariate model

The diagnostic plots demonstrate that the model *all_full* has a good fit (**Figures 4A–D**). In the multivariate model, the predicted Sqrt of CD4 cell count trend curve of the CM group remained consistently higher than that of the non-CM group, suggesting better overall immune recovery in the CM group. The trend curve of the multivariate model was smoother and closer to the center of the original data distribution, suggesting that the inclusion of additional covariates may have yielded a more robust estimation of population trends (**Figure 4E**). Specific values can be found in **Supplementary Tables S1**, **S2**. Even after controlling for other factors using multivariate LMEM analysis, the difference remained significant (estimate and 95% CI: 2.97, 1.91–4.02; *P <*0.05). CD4 cell counts increased over time, with greater increases observed in PLHIV with longer HIV infection duration, those receiving ART, and those with higher baseline CD4 cell counts, in addition to the observed effect of CM on CD4 cell count (*P <*0.05) (**Figure 4F**).

**Figure 4:**
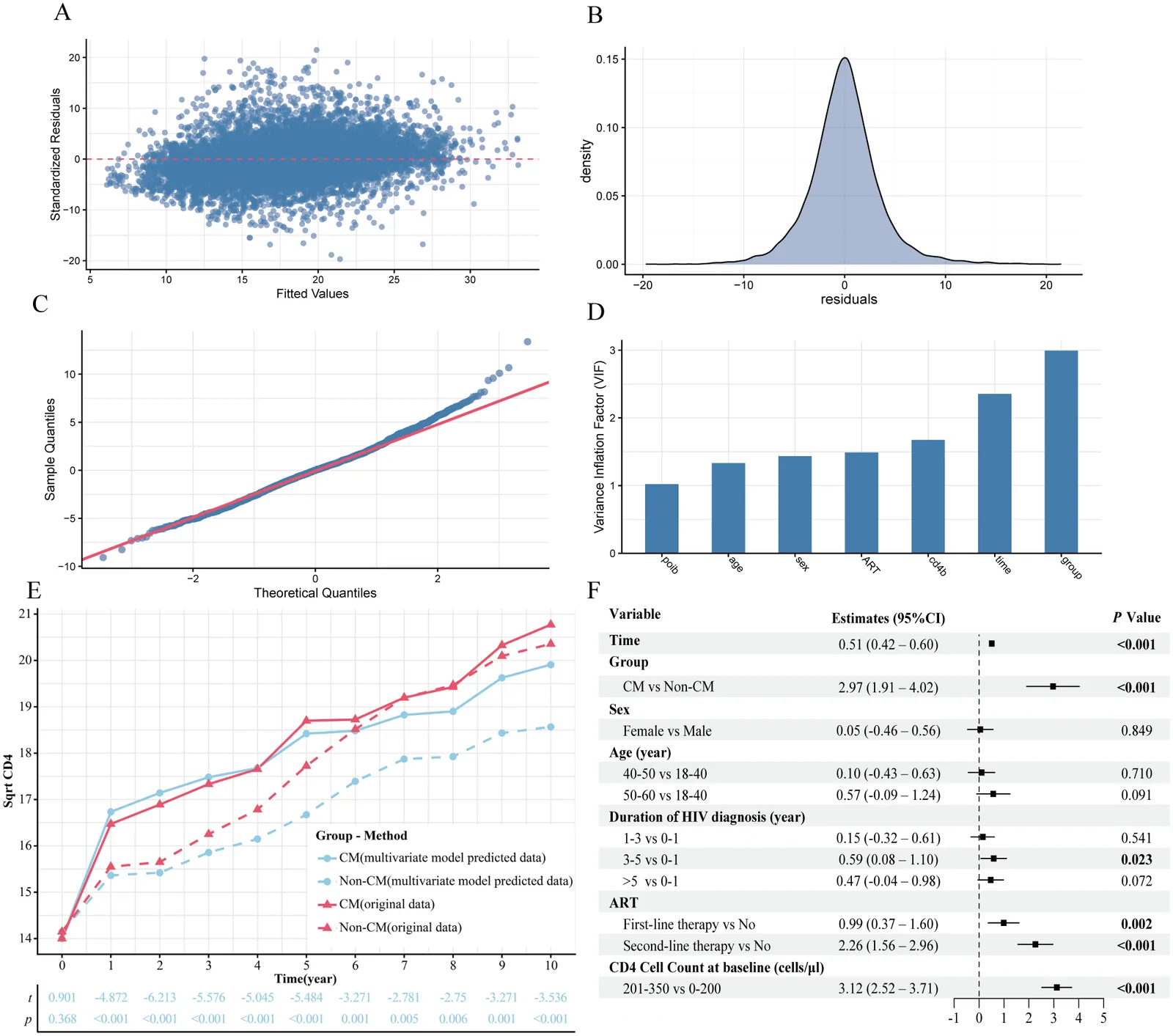
Diagnostic plots for the multivariate optimal mixed model. **(A)** the linear relationship diagnostic plot, **(B)** the normality plot for random effects, **(C)** the normality diagnosis of residuals and the normality quantile–quantile plot, **(D)** the collinearity diagnostic plot, **(E)** the Sqrt CD4 cell count annual trend plot, and **(F)** the results of the optimal multivariate model.

### Analysis of the effect of CM on the CD4 cell count of PLHIV by subgroup

Subgroup analysis was performed according to the classification of each variable, using the optimal multivariate LMEM described above and controlling for other variables, to analyze the effect of CM on the CD4 cell counts of subgroups of PLHIV. The results showed that CM could increase the CD4 cell count in almost all subgroups, except in the groups with HIV infection for 3–5 years or >5 years. There was an interaction between CM and sex (*P* = 0.009), and CM increased CD4 cell counts more in female than in male participants. There was an interaction between CM and baseline CD4 cell count (*P* < 0.001), and CM increased CD4 cell counts more in the subgroup with ≤200 cells/μL than in the subgroup with 200–350 cells/μL. The detailed results are summarized in **Figure 5**.

**Figure 5:**
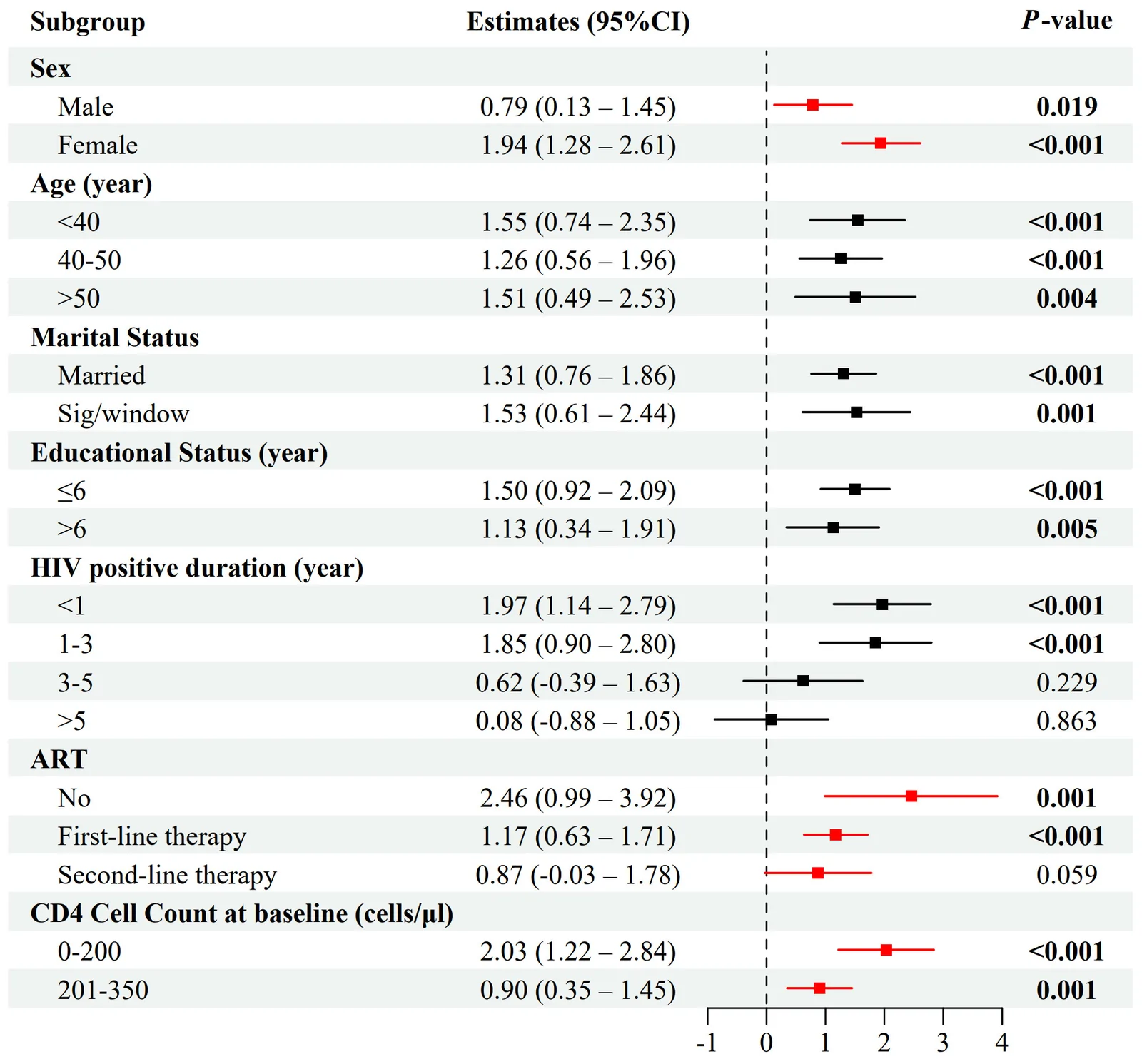
Subgroup analysis of the impact of CM on CD4 cell count. The dependent variable was the Sqrt of CD4 cell count. The non-CM group was deemed the control group. The LMEM was employed to calculate the estimated values after adjusting for other factors.

### Sensitivity analysis of the effect of CM on the CD4 cell count of by multivariate LMEMs

The sensitivity analysis was conducted using the multivariate LMEMs (*AR1_full, Var_full, ns_full*, and *all_full*). For the group effect (CM group versus non-CM group) and time effect, the 95% CIs of all models were located to the right of the null line (dotted line), suggesting that both the group effect and the time effect were positive and significant. For the interaction effect between group and time, except for the model *all_full*, the 95% CIs of the models were located to the left of the null line, indicating that the interaction effect remained stable in most model settings and was negative and significant, suggesting that CM intervention can effectively improve the CD4 cell count (**Figure 6**).

**Figure 6:**
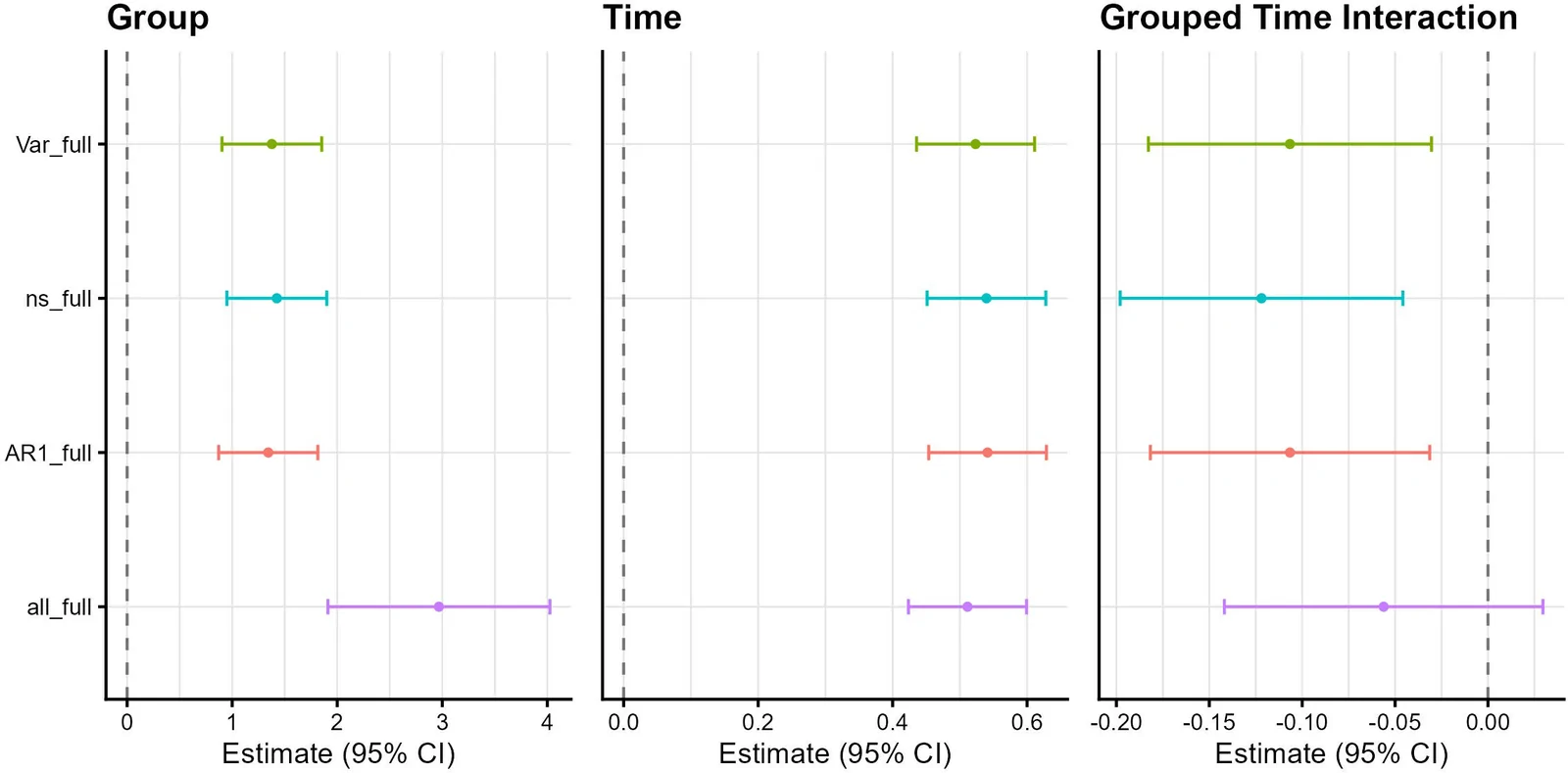
Sensitivity analysis of the impact of CM on CD4 cell count by multivariate LMEMs.

### Sensitivity analysis of the impact of CM on CD4 cell count after imputing the missing data of CD4 cell counts during the follow-up period

The presence of missing data on CD4 cell counts during the follow-up period is inevitable in a retrospective cohort study, which will affect the accuracy of the results. In this study, the reasons for the absence of CD4 cell counts during the follow-up period could not be determined (except for the cases of death), mainly because such information was not included in the conventional medical records. We conducted descriptive statistics on the missing data (**Supplementary Figures S2**–**S4**) and used multiple imputation and random forest models to fill in the missing data of CD4 cell counts during the follow-up period, respectively, and then used the model *all_full* to analyze the effect of CM on the CD4 cell count of PLHIV. The results showed that the CD4 cell count in the CM group increased more than in the non-CM group (**Figure 7A**; **Supplementary Figure S5A**; **Supplementary Tables S3**–**S6**). Even after controlling for other factors using multivariate LMEM analysis, the difference remained significant (estimate and 95% CI: 3.67, 2.58–4.75; *P <*0.05). CD4 cell counts increased over time, with greater increases observed in PLHIV with longer HIV infection duration, those receiving ART, and those with higher baseline CD4 cell counts, in addition to the observed effect of CM on CD4 cell count (*P <*0.05) (**Figure 7B**, **Figure S5B**). These results were consistent with the results of the multivariate analysis of the original data.

**Figure 7:**
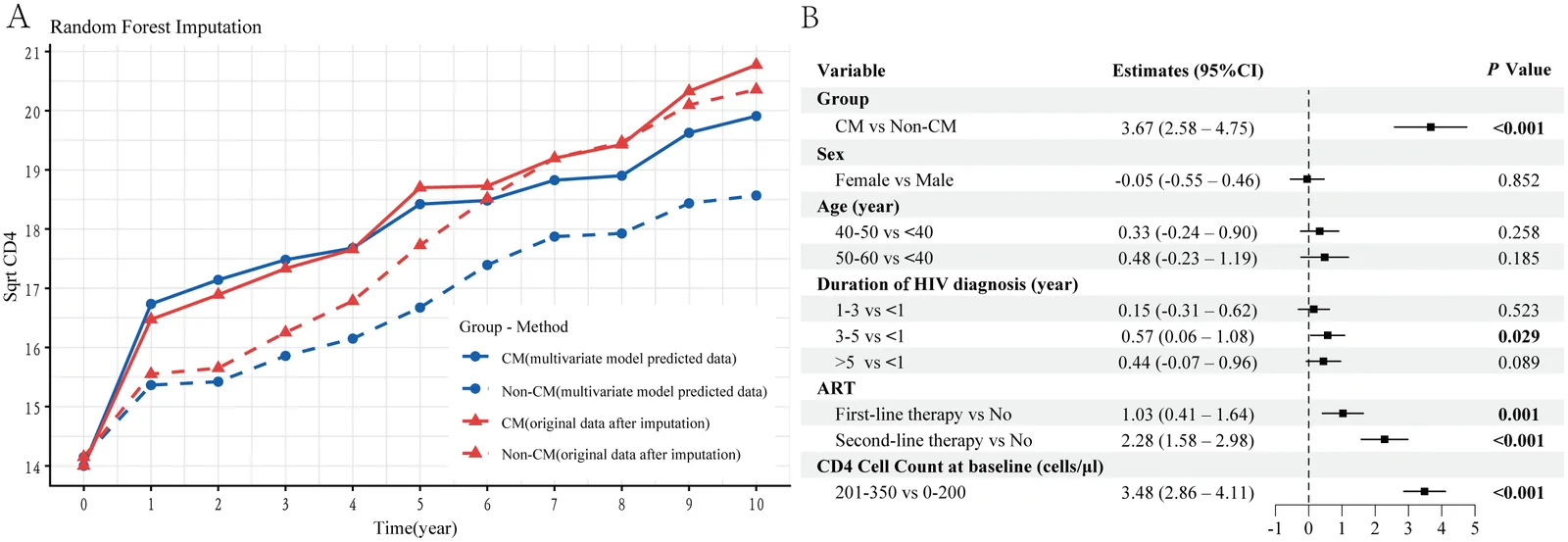
The impact of CM on CD4 cell count after random forest imputation of the missing data of CD4 cell counts during the follow-up period. **(A)** the Sqrt of CD4 cell count annual trend plot, and **(B)** the results of the optimal multivariate model.

## Discussion

In this study, we conducted a real-world study based on conventional medical records available in the NCMAIDSTP and NFATP of Henan Province, China, to quantitatively investigate the long-term effects of CM on the CD4 cell count among PLHIV. As a real-world study, it has inherent limitations such as inevitable selection bias and confounding bias. To ensure more robust conclusions from these real-world data, we used PS matching, a well-established technique to mitigate these biases, to control for confounding factors between groups in this study (24, 25). After PS matching, no differences in baseline characteristics were observed between the CM and non-CM groups. Real-world retrospective cohort studies often produce repeated-measures data, which violate the “independence” assumption of traditional linear regression owing to the correlation of multiple observations within an individual and feature random errors distributed at different levels. LMEMs describe individual heterogeneity by introducing random effects, and specify a flexible covariance structure to deal with intra-individual correlation, without assuming independence of observations or performing degree-of-freedom correction. In addition, based on likelihood estimation, this type of model can make full use of imbalanced data, and ensure the validity of parameter estimation and the accuracy of hypothesis testing while separating group-level fixed effects and individual-level random effects, enabling it to accurately fit the complex characteristics of real-world repeated measurement data (26, 27).

The CM intervention in this study was the Yiaikang capsule, which has been reported to increase CD4 cell count among PLHIV (28–30). According to CM theory, the herbs contained in Yiaikang capsules have invigorating effects on the spleen and qi while nourishing the blood, thereby enhancing zheng qi within the body. Although the mechanism by which Yiaikang capsules benefit PLHIV is not clear from a modern medical perspective, there are reports supporting their efficacy. One study reported that Yiaikang capsules can reduce the expression levels of ICAM-1 and LFA-1 in the CD4 cells of PLHIV, and that they may regulate the immune function of patients by stabilizing or increasing the number of CD4 cells (31). Two other studies found reduced expression levels of protein-regulated genes and a higher percentage of Th1 cells in PLHIV treated with Yiaikang capsules than in those who did not receive the capsules. *In vitro* experiments have shown that Yiaikang drug-containing serum can enhance the cytotoxic function of NK-92MI cells by inhibiting their apoptosis, downregulating the expression of inhibitory receptor NKG2A, and promoting the secretion of cytokine IFN-γ (32, 33). Our quantitative analysis supports the ability of the Yiaikang capsule to increase CD4 cell counts among PLHIV.

We found that the CD4 cell counts of PLHIV who received CM increased more rapidly in the first year of administration than the counts of PS-matched controls (after filling in the missing data of CD4 cell counts during the follow-up period using multiple imputation and random forest (34), the analysis was conducted, and the results were also consistent), consistent with previous studies (9). Numerous studies have reported a positive influence of CM on CD4 cell count in PLHIV (9, 15, 26, 31–33, 35), and most showed that CM increased the CD4 cell counts of PLHIV during the early stages of treatment. For instance, a study conducted in the Guangxi Province of China showed that the CD4 cell count of PLHIV increased quickly during the first 6 months of CM treatment (36). Our subgroup analysis showed that CM therapy was associated with a higher CD4 cell count in PLHIV among almost all subgroups, with a particular improvement in the group with a lower baseline CD4 cell count. Some studies have shown that PLHIV with a CD4 cell count <350 cells/μL have a higher mortality rate (37). Therefore, our data suggest that initiating CM therapy as early as possible could decrease mortality among PLHIV.

Many factors can influence CD4 cell count, such as sex, age, duration of ART treatment, baseline CD4 cell count, and adherence to ART (38–42). In this study, in addition to CM therapy, sex and baseline CD4 cell count were independent predictors of a higher CD4 cell count. Compared with male PLHIV, female PLHIV had a higher CD4 cell count after treatment, in agreement with the findings of previous studies (38–42). The greater improvement in CD4 cell counts in female PLHIV may be attributable to better behavioral habits, better adherence to treatment, and physiological characteristics such as lower levels of androgenic hormones that decrease thymic T-cell output (43). However, additional research will be needed to investigate the roles of these potential factors. In this study, baseline CD4 cell count positively correlated with later CD4 cell count, consistent with the findings of previous studies.

As a real-world retrospective cohort study, this research has some limitations. Firstly, data on some important factors that affect CD4 cell count were not collected, such as treatment compliance, HIV viral load, opportunistic infection status, coexisting diseases, and the administration of concurrent medications. Secondly, the characteristics of the study population were relatively uniform, with the majority being farmers, of Han ethnicity, and infected with HIV through blood transfusion. Meanwhile, the inevitable lack of CD4 cell counts during the follow-up period will affect the accuracy of the research results. Although we filled in the missing data for the sensitivity analysis, the data-missing patterns in the retrospective cohort study are complex. Therefore, more rigorous prospective studies need to be designed to confirm the efficacy of traditional CM for PLHIV.

## Conclusion

CM might be associated with maintaining higher CD4 counts on top of existing ART regimens, especially in those with a lower baseline CD4 cell count.

## Data Availability

The data analyzed in this study are subject to the following licenses/restrictions: The NFATP data were held by the Chinese Center for Disease Control and Prevention and the NCMATP data were held by the State Administration of Traditional Chinese Medicine. Restrictions apply to the availability of these data, which were used under license for the current study, and so are not publicly available. Requests to access these datasets should be directed to jentyking@126.com.
