# Supplementary material for: Exploring the impact of Chinese medicine on CD4 cell counts in people living with HIV by mixed-effects model: a retrospective cohort study based on propensity score matching
Source: Front Immunol. 2026 Sep 24;17:1863625. doi: 10.3389/fimmu.2026.1863625 (PMC13646109; doi:10.3389/fimmu.2026.1863625)
Supplement: Supplementary file 1 [file DataSheet1.pdf]

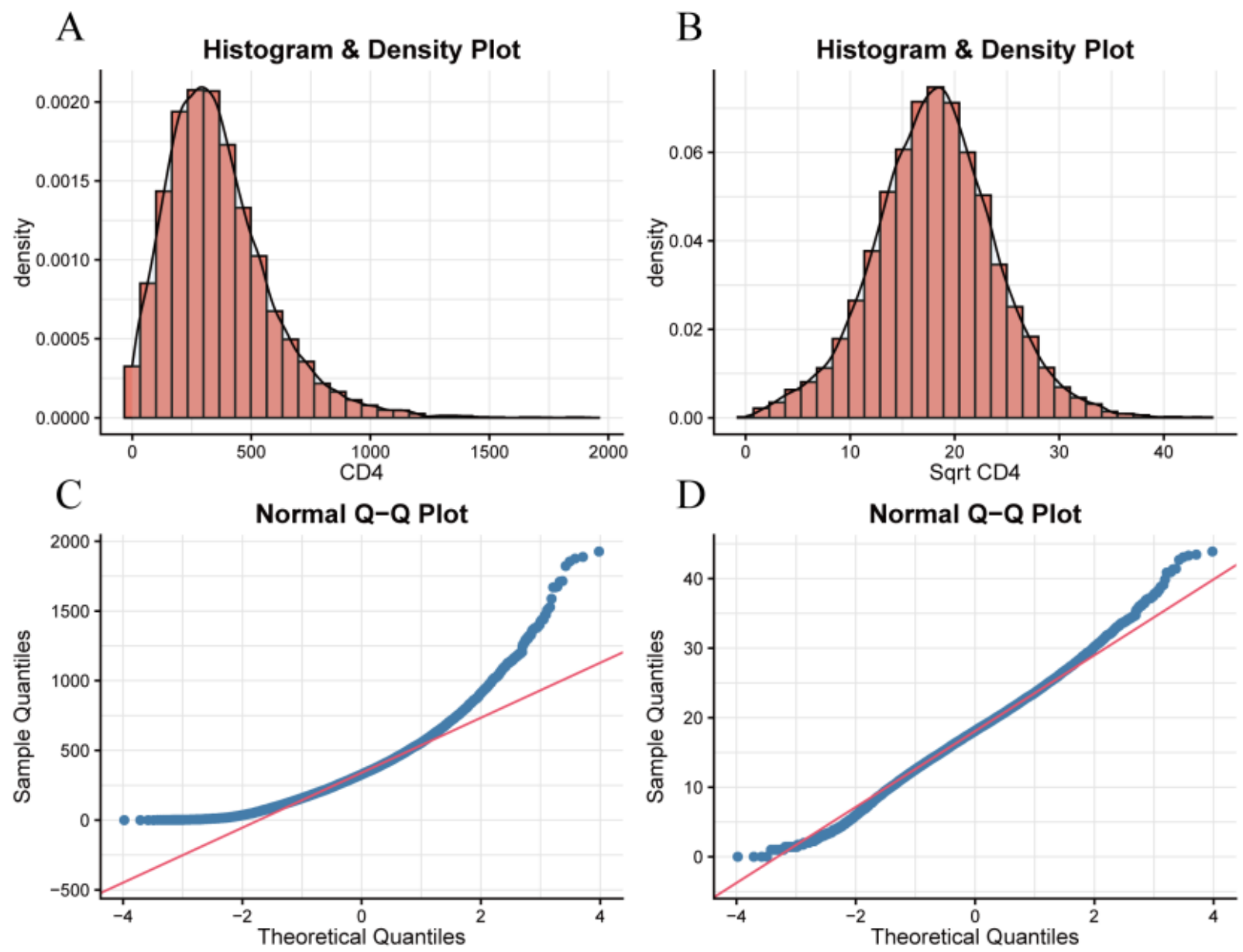

Figure S1. Normality test graph for CD4 cell count (A, C) and the square root of the CD4 cell count (B, D)

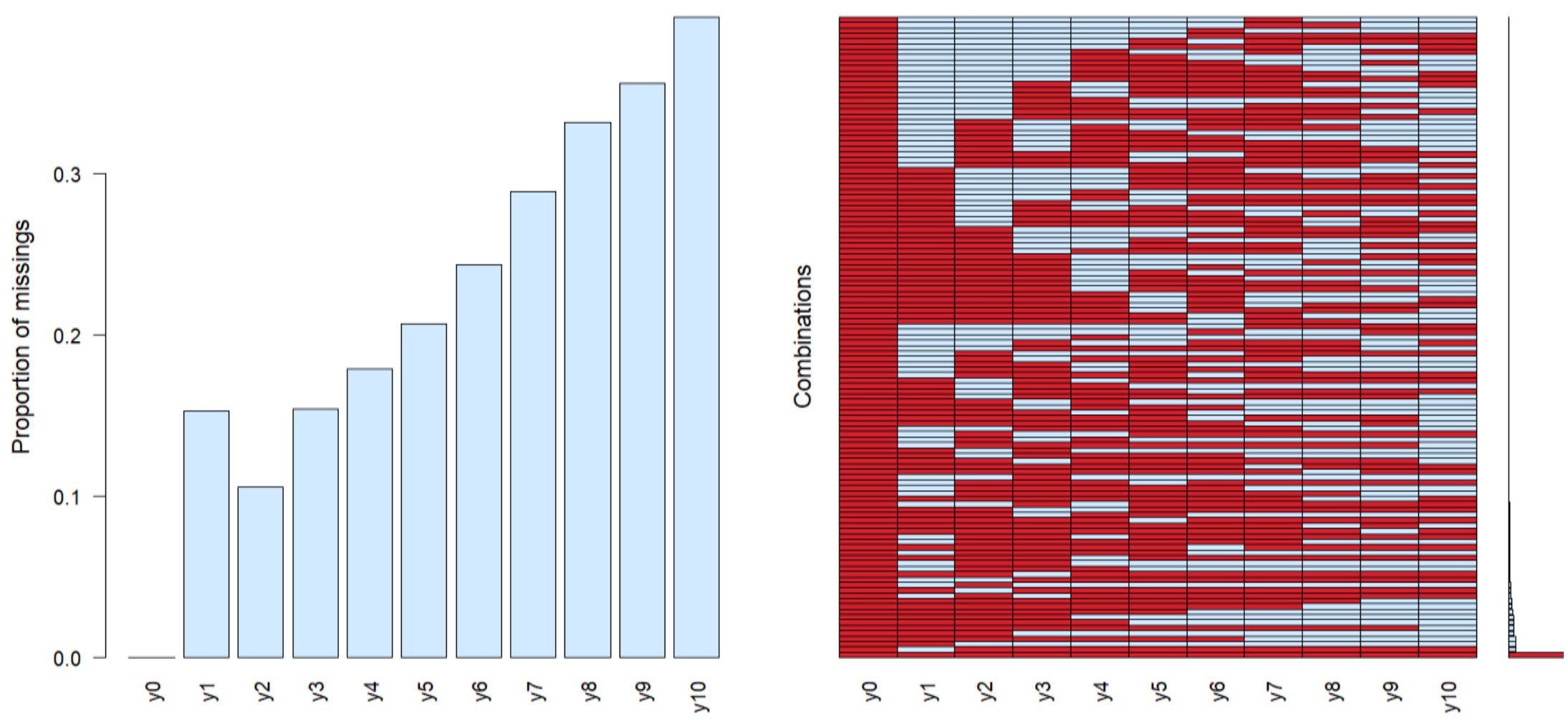

Figure S2. Proportion and pattern of missing CD4 counts per year

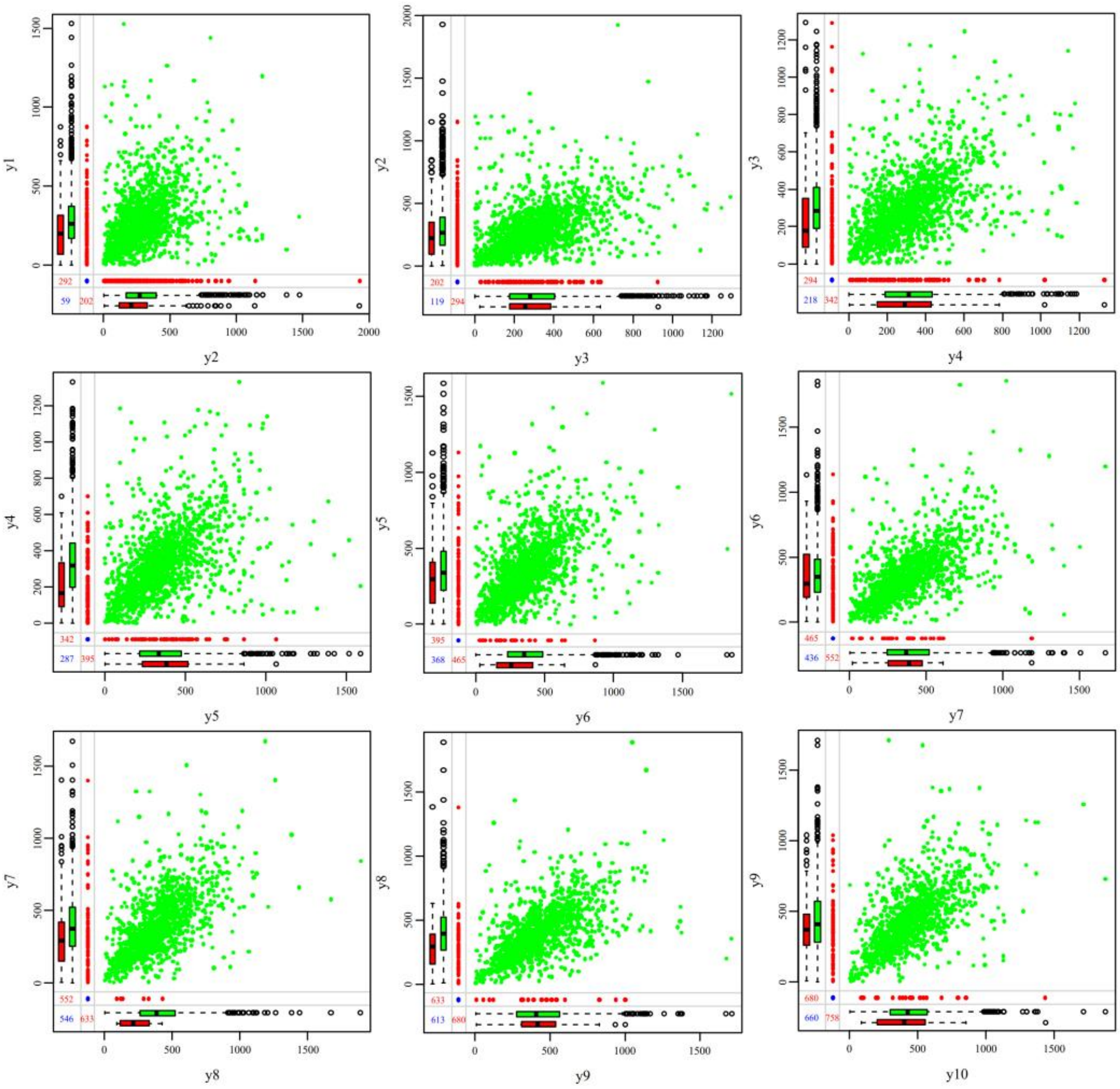

Figure S3. The relationship and distribution of the current year's CD4 cell count with the previous year's CD4 cell count (both axes represent CD4 cell count (cells/μl))

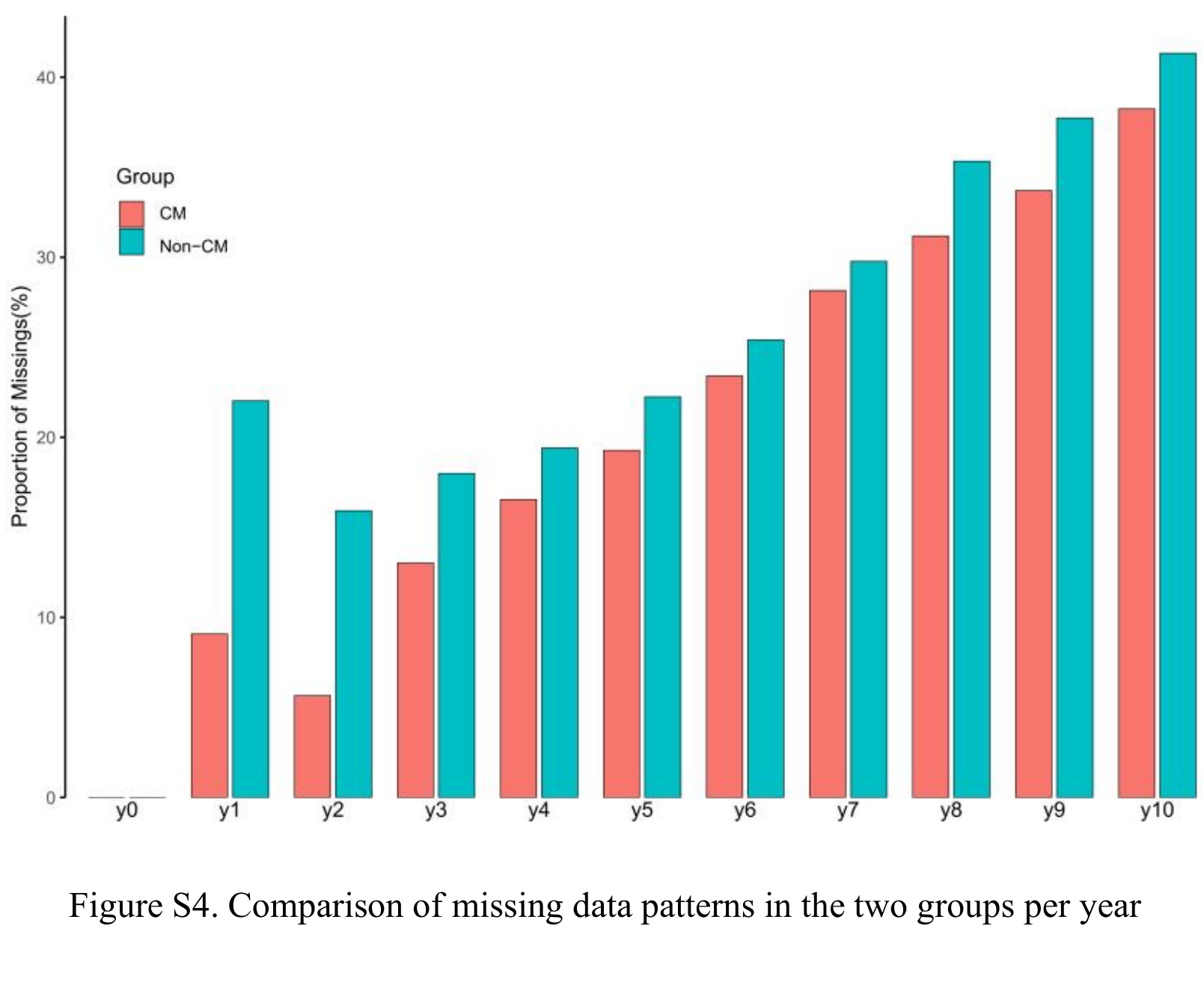

Figure S4. Comparison of missing data patterns in the two groups per year

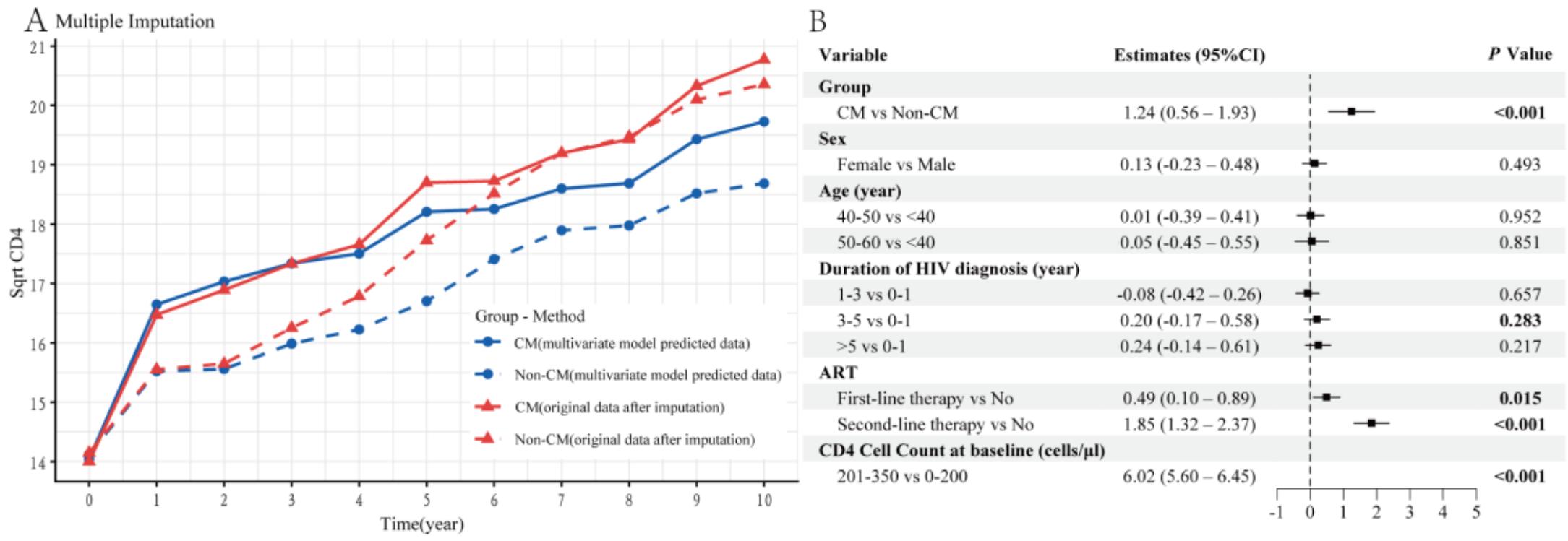

Figure S5. The effect of CM on CD4 cell count after multiple imputation of missing CD4 cell count data during the follow-up period and pooling of results using Rubin's rules (A) the Sqrt CD4 annual trend plot, (B) the results of the optimal multivariate model.

Table S1. The original Sqrt CD4 data of the two groups

time	CM		Non-CM		t-value	*P*-value
	Mean	95%CI	Mean	95%CI		
Baseline	14.00	13.77-14.23	14.15	13.92-14.37	0.901	0.368
Year 1	16.47	16.10-16.85	15.55	15.18-15.92	-3.427	0.001
Year 2	16.89	16.51-17.27	15.65	15.27-16.03	-4.551	<0.001
Year 3	17.33	16.95-17.72	16.25	15.89-16.62	-3.980	<0.001
Year 4	17.66	17.26-18.05	16.78	16.38-17.19	-3.041	0.002
Year 5	18.70	18.30-19.10	17.73	17.29-18.16	-3.255	0.001
Year 6	18.73	18.33-19.12	18.52	18.08-18.95	-0.703	0.482
Year 7	19.20	18.79-19.60	19.20	18.75-19.64	-0.006	0.995
Year 8	19.43	19.01-19.85	19.47	19.05-19.88	0.135	0.892
Year 9	20.33	19.91-20.75	20.10	19.65-20.54	-0.742	0.458
Year 10	20.77	20.33-21.21	20.36	19.87-20.84	-1.248	0.212

Table S2. Multivariate LMEM estimates Sqrt CD4 for the two groups based on the multivariate model predicted data

time	CM		Non-CM		t-value	*P*-value
	Mean	95%CI	Mean	95%CI		
Baseline	14.00	13.77-14.23	14.15	13.92-14.37	0.901	0.368
Year 1	16.74	16.33-17.14	15.36	14.96-15.77	-4.872	<0.001
Year 2	17.14	16.74-17.54	15.42	15.03-15.81	-6.213	<0.001
Year 3	17.48	17.05-17.91	15.86	15.45-16.27	-5.576	<0.001
Year 4	17.68	17.23-18.13	16.15	15.72-16.58	-5.045	<0.001
Year 5	18.42	17.95-18.89	16.67	16.22-17.12	-5.484	<0.001
Year 6	18.48	17.99-18.98	17.39	16.92-17.86	-3.271	0.001
Year 7	18.83	18.32-19.34	17.87	17.39-18.36	-2.781	0.005
Year 8	18.90	18.37-19.43	17.93	17.42-18.43	-2.750	0.006

Year 9	19.63	19.09-20.17	18.44	17.91-18.96	-3.271	0.001
Year 10	19.91	19.35-20.47	18.57	18.02-19.11	-3.536	<0.001

Table S3. The original Sqrt CD4 data after random forest imputation of the two groups

time	CM		Non-CM		t-value	*P*-value
	Mean	95%CI	Mean	95%CI		
Baseline	14.00	13.77-14.23	14.15	13.92-14.37	0.901	0.368
Year 1	16.47	16.10-16.85	15.55	15.18-15.92	-3.427	0.001
Year 2	16.89	16.51-17.27	15.65	15.27-16.03	-4.551	<0.001
Year 3	17.33	16.95-17.72	16.25	15.89-16.62	-3.980	<0.001
Year 4	17.66	17.26-18.05	16.78	16.38-17.19	-3.041	0.002
Year 5	18.70	18.30-19.10	17.73	17.29-18.16	-3.255	0.001
Year 6	18.73	18.33-19.12	18.52	18.08-18.95	-0.703	0.482
Year 7	19.20	18.79-19.60	19.20	18.75-19.64	-0.006	0.995
Year 8	19.43	19.01-19.85	19.47	19.05-19.88	0.135	0.892
Year 9	20.33	19.91-20.75	20.10	19.65-20.54	-0.742	0.458
Year 10	20.77	20.33-21.21	20.36	19.87-20.84	-1.248	0.212

Table S4. Multivariate LMEM estimates Sqrt CD4 for the two groups based on the multivariate model predicted data after random forest imputation

time	CM		Non-CM		t-value	*P*-value
	Mean	95%CI	Mean	95%CI		
Baseline	14.00	13.77-14.23	14.15	13.92-14.37	0.901	0.368
Year 1	16.74	16.33-17.14	15.36	14.96-15.77	-4.872	<0.001
Year 2	17.14	16.74-17.54	15.42	15.03-15.81	-6.213	<0.001
Year 3	17.48	17.05-17.91	15.86	15.45-16.27	-5.576	<0.001
Year 4	17.68	17.23-18.13	16.15	15.72-16.58	-5.045	<0.001
Year 5	18.42	17.95-18.89	16.67	16.22-17.12	-5.484	<0.001
Year 6	18.48	17.99-18.98	17.39	16.92-17.86	-3.271	0.001
Year 7	18.83	18.32-19.34	17.87	17.39-18.36	-2.781	0.005
Year 8	18.90	18.37-19.43	17.93	17.42-18.43	-2.750	0.006
Year 9	19.63	19.09-20.17	18.44	17.91-18.96	-3.271	0.001
Year 10	19.91	19.35-20.47	18.57	18.02-19.11	-3.536	<0.001

Table S5. The original Sqrt CD4 data of the two groups after multiple imputation and pooling of results using Rubin's rules

time	CM		Non-CM		t-value	*P*-value
	Mean	95%CI	Mean	95%CI		
Baseline	14.00	13.77-14.23	14.15	13.92-14.37	0.901	0.368
Year 1	16.47	16.10-16.85	15.55	15.18-15.92	-3.427	0.001
Year 2	16.89	16.51-17.27	15.65	15.27-16.03	-4.551	<0.001
Year 3	17.33	16.95-17.72	16.25	15.89-16.62	-3.980	<0.001

Year 4	17.66	17.26-18.05	16.78	16.38-17.19	-3.041	0.002
Year 5	18.70	18.30-19.10	17.73	17.29-18.16	-3.255	0.001
Year 6	18.73	18.33-19.12	18.52	18.08-18.95	-0.703	0.482
Year 7	19.20	18.79-19.60	19.20	18.75-19.64	-0.006	0.995
Year 8	19.43	19.01-19.85	19.47	19.05-19.88	0.135	0.892
Year 9	20.33	19.91-20.75	20.10	19.65-20.54	-0.742	0.458
Year 10	20.77	20.33-21.21	20.36	19.87-20.84	-1.248	0.212

Table S6. Multivariate LMEM estimates Sqrt CD4 for the two groups based on the multivariate model predicted data after multiple imputation and pooling of results using Rubin's rules

time	CM		Non-CM		t-value	*P*-value
	Mean	95%CI	Mean	95%CI		
Baseline	14.04	13.74-14.34	14.12	13.85-14.40	-0.407	0.684
Year 1	16.65	16.31-16.98	15.52	15.20-15.84	4.918	<0.001
Year 2	17.03	16.68-17.39	15.56	15.21-15.91	6.024	<0.001
Year 3	17.34	16.94-17.74	15.98	15.60-16.37	4.950	<0.001
Year 4	17.50	17.07-17.94	16.23	15.81-16.65	4.349	<0.001
Year 5	18.21	17.75-18.66	16.70	16.26-17.14	4.879	<0.001
Year 6	18.25	17.78-18.72	17.41	16.96-17.86	2.655	0.008
Year 7	18.60	18.12-19.08	17.90	17.43-18.36	2.172	0.030
Year 8	18.69	18.19-19.18	17.98	17.50-18.46	2.125	0.034
Year 9	19.43	18.92-19.94	18.52	18.02-19.02	2.643	0.008
Year 10	19.73	19.19-20.26	18.69	18.16-19.22	2.859	0.004
